# Phrynoderma in a patient with megaloblastic anemia

**DOI:** 10.4103/0301-4738.73704

**Published:** 2011

**Authors:** Vishal Sharma, Mukul P Agarwal, Subhash Giri

**Affiliations:** Department of Medicine, University College of Medical Sciences (University of Delhi) & GTB Hospital, Delhi, India

Dear Editor,

We read with interest the case report linking phrynoderma and night blindness by Murthy *et al*.[1] The authors have done well to document the coexistence.[1] We would like to share our experience with a similar case.

**Figure 1:**
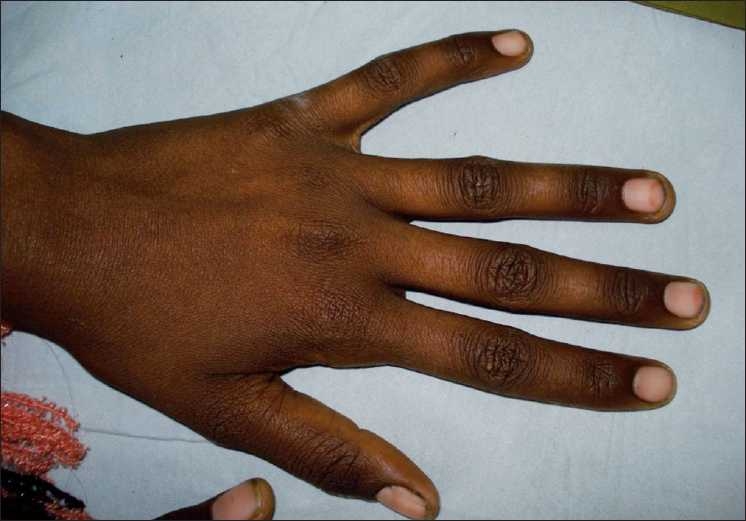
Hperpigmented knuckles

**Figure 2:**
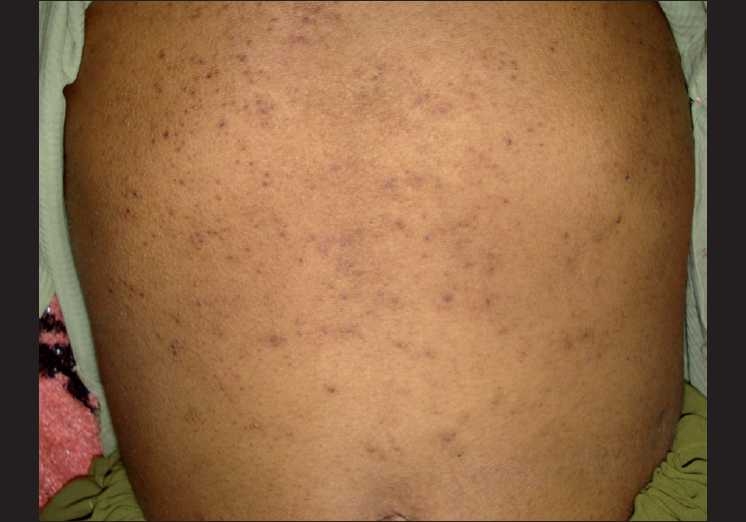
Toad-like skin over trunk

A 15-year-old girl presented with history of anorexia, generalized fatigue, decreased night vision and skin lesions. She had been ill for around 6 months and had previously received a blood transfusion elsewhere. She denied any history of fever or any other chronic illness. She was pale and had hyperpigmented knuckles [Fig. 1]. The patient had a papular skin rash suggestive of toad skin on her elbows and abdomen [Fig. 2]. Sclera had a lemony tinge. The conjunctiva appeared dry but there was no evidence of any Bitot spots or corneal lesion. Palpation of abdomen revealed an enlarged liver and spleen. The hemogram was suggestive of macrocytic anemia (hemoglobin 7.2 g/dL, total leukocyte count 3200/μL, platelets 88,000/μL, mean corpuscular volume 102 fL). Peripheral smear revealed macrocytes and hypersegmented neutrophils. Serum vitamin B-_12_ levels were reduced to 112 pg/mL (normal range: 279–996 pg/mL). Folate levels were also reduced to 2 ng/mL (normal range: 5.4–18.0 ng/mL). Bone marrow examination was consistent with the diagnosis of megaloblastic anemia. Liver functions revealed slightly elevated serum bilirubin (3.2 mg/dL) with normal enzymes and serum albumin. The patient was put on replacement with cobalamin, folic acid and vitamin A. The anemia and the skin lesions resolved over 6 months of follow-up.

We present this case for two reasons. First, the case demonstrates coexistence of phrynoderma and decreased nocturnal vision. Also, the coexistence of features of vitamin A, folate and B12 deficiency existed in this case. It is important because of coexistence of both water and fat soluble vitamin deficiency. It may be noted that current belief is that multiple nutrient deficiencies coexist to result in phrynoderma.[2] It is therefore of importance that patients with phrynoderma be evaluated not just for vitamin A deficiency but also for deficiency of vitamins B, C and E.
